# Anti-Tumor Efficiency of Perillylalcohol/β-Cyclodextrin Inclusion Complexes in a Sarcoma S180-Induced Mice Model

**DOI:** 10.3390/pharmaceutics13020245

**Published:** 2021-02-10

**Authors:** Allan A. Rezende, Rafael S. Santos, Luciana N. Andrade, Ricardo G. Amaral, Matheus M. Pereira, Cristiane Bani, Mo Chen, Ronny Priefer, Classius F. da Silva, Ricardo L. C. de Albuquerque Júnior, Eliana B. Souto, Patrícia Severino

**Affiliations:** 1 University of Tiradentes (Unit), Postgraduate Program Industrial Biotechnology, and Health and Enviroment. Av. Murilo Dantas, 300, 49010-390 Aracaju, Brazil; allan_a.rezende@hotmail.com (A.A.R.); rafa.au@icloud.com (R.S.S.); ricardo.patologia@uol.com.br (R.L.C.d.A.J.); 2 Institute of Technology and Research (ITP), Av. Murilo Dantas, 300, 49010-390 Aracaju, Brazil; 3 Department of Physiology, Federal University of Sergipe, São Cristovão, 49100-000 Sergipe, Brazil; luciana.nalone@hotmail.com (L.N.A.); ricardoamaral23@hotmail.com (R.G.A.); crisbani@gmail.com (C.B.); 4 CICECO, Aveiro Institute of Materials, Department of Chemistry, University of Aveiro, 3810-193 Aveiro, Portugal; matheus.pereira@ua.pt; 5 Department of Gynecology, Obstetrics & Gynecology, Obstetrics and Gynecology Hospital of Fudan University, Shanghai 200011, China; chenmo7109@fckyy.org.cn; 6 School of Pharmacy, Massachusetts College of Pharmacy and Health Sciences, 179 Longwood Avenue, Boston, MA 02115, USA; ronny.priefer@mcphs.edu; 7 Institute of Environmental, Chemical and Pharmaceutical Sciences, Federal University of São Paulo, 09972-270 Diadema, São Paulo, Brazil; classiusferreira@yahoo.com.br; 8 Faculty of Pharmacy, University of Coimbra, Pólo das Ciências da Saúde, Azinhaga de Santa Comba, 3000-548 Coimbra, Portugal; 9 CEB—Centre of Biological Engineering, University of Minho, Campus de Gualtar, 4710-057 Braga, Portugal; 10 Tiradentes Institute, 150 Mt Vernon St, Dorchester, MA 02125, USA; 11 Center for Biomedical Engineering, Department of Medicine, Brigham and Women& Hospital, Harvard Medical School, 65 Landsdowne Street, Cambridge, MA 02139, USA

**Keywords:** perillyl alcohol, cyclodextrin, inclusion complex, anti-cancer, sarcoma

## Abstract

The low solubility and high volatility of perillyl alcohol (POH) compromise its bioavailability and potential use as chemotherapeutic drug. In this work, we have evaluated the anticancer activity of POH complexed with β-cyclodextrin (β-CD) using three complexation approaches. Molecular docking suggests the hydrogen-bond between POH and β-cyclodextrin in molar proportion was 1:1. Thermal analysis and Fourier-transform infrared spectroscopy (FTIR) confirmed that the POH was enclosed in the β-CD cavity. Also, there was a significant reduction of particle size thereof, indicating a modification of the β-cyclodextrin crystals. The complexes were tested against human L929 fibroblasts after 24 h of incubation showing no signs of cytotoxicity. Concerning the histopathological results, the treatment with POH/β-CD at a dose of 50 mg/kg promoted approximately 60% inhibition of tumor growth in a sarcoma S180-induced mice model and the reduction of nuclear immunoexpression of the Ki67 antigen compared to the control group. Obtained data suggest a significant reduction of cycling cells and tumor proliferation. Our results confirm that complexation of POH/β-CD not only solves the problem related to the volatility of the monoterpene but also increases its efficiency as an antitumor agent.

## 1. Introduction

Perillyl alcohol (POH) is a monoterpene of essential oils found in large quantities in cherries, lavender, mint, sage, sagebrush, perilla, holy grass (lemongrass), ginger bergamot, ginger leaves, *Armenian cumin* and seeds of celery. It has been widely used for antineoplastic treatment [1,2]. The conjugation of POH with temozolomide was shown to block the induction of the pro-angiogenic process, thus demonstrating to be a potential inhibitor of tumor progression and recurrence in glioma stem cells [3]. In therapy against glioblastoma lineage, the efficacy of POH administered intranasally has also been described [4,5]. Faria et al. administered POH by intranasal route in terminally ill patients with glioblastoma and cerebral adenocarcinoma metastasis (stage IV), yielding a 6-month survival rate [6]. In another work, Song et al. described the selectivity of POH against cancer cell lines with the induction of apoptosis, cytotoxicity and effectiveness in compromising the tumor mitochondrial membrane [7]. These findings make this compound as the most effective of the series against all cell lines [8,9]. POH is thus recognized as a potent antitumor activity with enormous potential in chemotherapy. However, POH shows low solubility which compromises its bioavailability, being also associated with a series of gastrointestinal side effects when orally administered.

Cyclodextrins (CDs) have been used to form host-guest inclusion complexes to improve solubility and bioavailability of drugs [10], to which CDs form non-covalent complexes [11,12]. In an aqueous solution, the slightly non-polar cavity is occupied by water molecules that are energetically unfavorable. Given the nature of the polar-non-polar interaction, water can be relatively easily replaced by a substrate that is less polar than water. Thus, the driving force for complexation is through the replacement of high-enthalpy water molecules with appropriate substrates [13]. This inclusion does alter drug characteristics significantly by improving bioavailability, solubility, stability and providing controlled release. CDs are widely used as drug carriers in cancer therapy favoring permeation and retention of the drug inside the cell cancer [14]. CDs have been complexed with chemotherapeutic agents against a variety of cancers, for example, bladder [15], colorectal [16], skin [17], lung [18], intestinal [19], colon [20], prostate [21] and liver [22].

There is an increasingly interest in exploring innovative technologies to improve the solubility of drugs [23,24]. The use of cyclodextrins as inclusion complexes for such substances is one of the most widely used methods that have been exploited for a range of compounds [25]. Besides, it modifies the drug release profile [26]. In the present work, β-CD has been selected on the basis of reports successfully describing the synthesis of inclusion complexes [27,28,29]. Advantages of complexing compounds with β-CDs are described for non-polar natural products, such as terpenes and essential oils [30]. To our knowledge, our work is the first report describing the complexation of POH with β-CD and its characterization for potential use as a chemotherapeutic strategy.

The aim of this work has been the synthesis of POH/β-cyclodextrin inclusion complexes by different approached, their characterization by Fourier transform infrared (FTIR) and scanning electron microscopy (SEM), molecular docking and thermal analysis to describe the interaction between the drug and the host, as well as their profiling with respect to cell viability in human fibroblast line L929 and anticancer activity in a sarcoma S180 induced mice model.

## 2. Materials and Methods

### 2.1. Materials

Perillyl alcohol (POH) (CAS: 18457-55-1, 98.8% purity) and β-cyclodextrin (β-CD) (CAS: 7585-39-9, ≥97%, molecular weight 1135.01) were purchased from Sigma-Aldrich (St. Louis, MO, USA). All other reagents were also bought from Sigma-Aldrich (St. Louis, MO, USA). The water was supplied by Milli-Q (Millipore, Burlington, MA, USA).

### 2.2. Molecular Docking

Auto-dock vina analyzed molecular interactions between β-CD and POH. The molecular structures created through DS Visualizer to employ molecular coupling. Auto DockTools (ADT, La Jolla, CA, USA) was used to prepare input files by fusing nonpolar hydrogen atoms, adding partial charges and atom types. ADT generated rigid binder root and receiver binder root, defining all possible rotary links as twist-active. The grid at the center of mass (x, y and z axes, respectively) to cover the entire β-CD interaction surface was 40 × 40 × 40 Å-10 different models underwent an assessment to find the binding model with the lowest free energy [31,32].

### 2.3. Preparation Perillyl Alcohol/β-CDs Complexes

The complexation was done by physical mixture, co-evaporation and malaxation using 1:1 molar ratio (POH:β-CD), after selection among the tested combinations (1:0.25, 1:1 and 1:2) as previously described [33]. The co-evaporation (CO) technique mixed β-CD and POH, followed by the progressive addition of distilled water to the formation of a solution and malaxation (MA), consisted of the mixture of β-CD, POH and 20 mL of water using magnetic stirring for 36 h. Finally, drying was carried out using a vacuum pump. The physical mixture (PM) was used as control and consisted of lightly homogenized both components with a mortar. All samples were hermetically stored until the characterization [34].

### 2.4. Thermal Analysis by DSC and TG/DTG

Differential scanning calorimetry (DSC) curves (DSC 2010 TA Instruments, New Castle, DE, USA) utilized a temperature range from 25–500 °C, under N_2_ atmosphere, with a 50 mL/min gas flow rate and a 10 °C/min heating ratio. The sample holder used was aluminum, containing 2 mg of the sample. Thermogravimetric curves were obtained from Shimadzu TG-60 with temperature ranging from 25 to 900 °C, under nitrogen (N_2_) atmosphere, with a gas flow rate of 50 mL/min and a 10 °C/min heating ratio. All analyses were performed using a platinum sample holder containing 7 mg of the sample.

### 2.5. Fourier-Transform Infrared Spectroscopy (FTIR)

FTIR spectrum was obtained using Agilent Caryn 630 FTIR (Agilent Technologies, Santa Clara, CA, USA) with attenuated total reflectance device (Miracle ATR, Pike Technologies Spectroscopic Creativity, Madison, WI, USA) using selenium crystal. The spectra were obtained in the 400–4000 cm^−1^ wavenumber range with resolution < 2/cm and processed for automatic data acquisition by Agilent Microlab PC software (Agilent Technologies, Santa Clara, CA, USA).

### 2.6. Scanning Electron Microscopy (SEM)

The morphological characteristics of the POH, β-CD and POH:β-CD (1:1) produced by CO were analyzed by SEM (Hitachi TM 3000, Tokyo, Japan). The samples were mounted on aluminum stubs, subjected to gold beam metallization and analyzed under an operated 8 Kv voltage acceleration electron microscope [35].

### 2.7. Viability Assay

Viability assay was performed with human fibroblast line L929 based on the ISO 10993–5 [36]. L929 cells (ATCC, LGC Standards S.L.U., Barcelona, Spain) were seeded in 96-well culture plates (2 × 10^4^ cells/well) and cultured in Dulbecco’s Modified Eagle’s Medium (DMEM) composed of NaHCO_3_ (1.2 g/L), ampicillin (0.025 g/L), streptomycin (0.1 g/L), supplemented with 10% fetal bovine serum (SFB). The cells were subjected to different concentrations (0–150 µg/mL) of the samples for 24 h at 37 °C and 5% CO_2_. Cell viability was assessed using the colorimetric method with Methyl-thiazolyl-tetrazolium (MTT). A solution of 0.025 g diluted in 50 mL of PBS was placed in contact with the cells, which were incubated at 37 °C for 3 h. After removing the MTT, dimethyl sulfoxide (DMSO) was added for 10 min for the solubilization of the crystals of the tetrazolium salt and soon afterward the optical density (DO) was read in an automated plate reader (ELISA) at a length of 570 nm wave. The tests were performed in quadruplicate and normalized.

### 2.8. Antitumor Activity

#### 2.8.1. Animals

First, the experimental protocol was submitted and approved by the Animal Care and Use Committee (CEUA) at Tiradentes University under registration number 041217. Twenty Swiss mice (females, weight varying between 25 and 30 g) were obtained from the central biotherium of Tiradentes University. The animals were housed and kept under a temperature-controlled room (22–25 °C), with 12:12 h light-dark cycle and free access to food and water. Sarcoma 180 (S180) cells were obtained from the Federal University of Sergipe. The donor animal was anesthetized via intraperitoneal and euthanized in a CO_2_ chamber. A volume of 0.5 mL of ascitic tumor cells was then aspirated from the animal’s abdominal cavity so that viable cells were selected using the trypan blue exclusion method [37]. A cells density of 2 × 10^6^ cells/0.5 mL/mouse was inoculated into the recipient animals, subcutaneously, in the animal’s left axillary region [38,39]. After 24 h of inoculation, treatments were started to assess the effect of drugs on tumor growth, one application a day for 7 consecutive days. The animals were split in 5 groups (*n* = 6) denominated vehicle (saline 0.9%), 5-FU (25 mg/kg/day) and β-CD:POH (50, 100 and 200 mg/kg/day). Administration of drugs and saline solution was performed daily by intraperitoneal route. After 24 h the last day of treatment, the animals were euthanized by induction in a CO_2_ chamber and the tumors were removed. The specimens were fixed in 10% formaldehyde for 24 h and followed a sequence of dehydration steps in increasing concentrations of alcohol, diaphanized in xylol and embedded in paraffin. Subsequently, serial histological sections (5 µm thick) of each specimen were obtained to perform the histochemical and immunohistochemical techniques. Histopathological analysis of the tumors consisted of each specimen were stained by routine histochemical technique (HE). In these, variables inherent to tumor cells (e.g., cell morphology, degree of cytological atypia and mitotic activity) were analyzed in a descriptive manner and related to tumor/host interaction (e.g., areas of necrosis coagulative, intraparenchymal and peritumoral inflammatory infiltrate and tumor boundaries).

#### 2.8.2. Proliferative Index in Tumor Cells Using In Situ Immunodetection of Ki67 Antigen

Histological sections with 3 µm thick were mounted on glass slides previously salinized and subjected to immunohistochemistry reaction using the indirect streptavidin-biotin method. The sections were deparaffinized in xylol and washed in decreasing concentrations of ethyl alcohol (100%, 95%, 90%, 80% and 70%). Antigenic recovery was performed by immersing the sections in a citrate solution, heated for 20 min in a microwave. The marking of the Ki-67 antigen was performed by incubation with the rabbit anti-mouse monoclonal antibody MIB-1 (Dako, Glostrup, Denmark, at 1:50 dilution), for 30 min. The reaction was revealed using diaminobenzidine (DAB, Ventana Medical Systems, Tucson, AZ, USA) and stained against Meyer’s Hematoxylin. Both steps were performed at an interval of four min each. The positive control was performed with human tonsil and negative control; the primary antibody was replaced by phosphate-buffered saline in the reaction. The positivity of the immunostaining was evidenced by the visualization of a cellular precipitate in a brownish tone. Thus, cells whose nuclei are immunostained (stained in brown), regardless of the intensity of the staining, were considered positive.

## 3. Results and Discussion

Molecular docking suggests bond simulations between molecules, that is, an efficient link between a macromolecule (receptor) and a small molecule (ligand), in this case between β-CD and POH, respectively, obtained from simulations/homology modeling. Molecule bound profiles have a particular feature of screening virtual libraries of substance-like molecules in order to obtain clues for further drug development. Docking was used to predict the conformation of POH/β-CD inclusion complex [40] and to estimate bound conformations and binding affinity [41].

**Figure 1:**
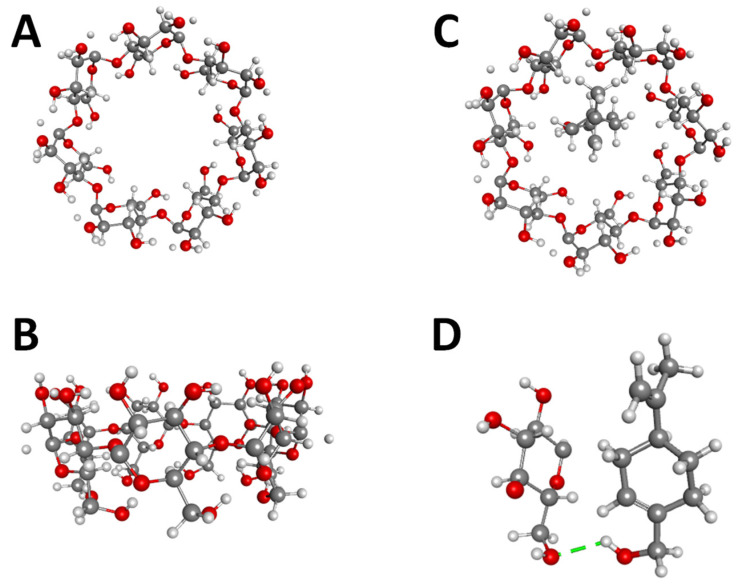
(**A**) and (**B**) β-CD structure, (**C**) and (**D**) molecular docking between POH and β-CD.

Figure 1 shows the β-CD structure (A and B) and the inclusion of complex hydrogen bonds (C and D) that occur between POH and β-CD. The best binding position and anchor affinities, interaction molecules, interaction type and geometry distance (Å) are shown in Table 1. The hydrogen-bonds result from the presence of OH groups within the POH structure. This result suggests a stronger bond due to the lower affinity energy [42]. These established hydrogen bonds also suggest that while CH_2_ and CH_3_ groups remain exposed, the main POH molecule is attached to the internal structure of β-CD. Molecular docking also allowed us to check the anchoring capacity of different POH amounts linked to β-CD, from which the 1:1 molar ratio was shown to be the most stable structure, thus selected for further studies.

POH, β-CD and the complexes obtained upon physical mixture (PM), malaxation (MA) and co-evaporation (CO) were subjected to the analysis by DSC and TG/DTG. Figure S1 (Supplementary material) shows the DSC curve of β-CD depicting two endothermic peaks; the first recorded between 37.44 and 108.35 °C (−427.66 J/g) and the second between 300.45 and 346.99 °C (−578.10 J/g), attributed to water evaporation and decomposition, respectively. POH displayed a broad endothermic peak between 26.02 and 94.42 °C (−257.22 J/g), corresponding to its volatilization.

The DSC curves of the raw materials (POH and β-CD) compared to those obtained by the analyses of the complexes (CO, PM and MA) confirm that the best interaction between the POH and β-CD was obtained with CO methodology; the POH reduction of the melting endotherm (73.14 to 127.40 °C, −29.41 J/g) was more significant compared to other methodologies (PM and MA). PM and MA show two endothermic peaks similar to the raw material’s profile, indicating the presence of the free form of β-cyclodextrin. The CO (POH:β-CD, 1:1) thermogram suggests that this method offers a more efficient complexation, attributed to the incorporation of POH in the internal cavity of β-CD. The melting event of POH shows a reduction in its peak. Additionally, during complexation, the water molecules present in the β-CD are removed and replaced by the guest molecule, thus favoring the formation of the complex and changing the thermoanalytical curve of the systems. Compared to CO, MA and PM approaches showed a smaller reduction in the melting peaks.

The formation of the POH/β-CD inclusion complex was also studied by TG/DTG. Figure S2 (Supplementary material) shows the TG curves of POH and β-CD raw materials and the complexes obtained by CO, PM and MA. From the depicted data, the POH suffers decomposition and volatilizes before 100 °C is reached. β-CD shows a thermoanalytical profile, which can be split into four parts. The first depression involves water loss (12.97%). Between 100 and 250 °C, the TG curve is flat and a low mass loss is detected (0.32%) and the thermal decomposition (around 89.70%) occurs between 250 to 500 °C (confirmed by DTG). Then continuous carbonization occurs in a wide temperature range that is, from 500 °C to about 700 °C (Δ*m* = 4.97%). A similar result has been described by Menezes et al. [43]. A similar profile was observed for the PM, which shows the overlap of individual components indicating low interaction (and therefore low degree of interaction) between POH and β-CD. In the TG analyses of complexes, the first mass loss event can be attributed to the water evaporation. A reduction of the intensity of this step can be seen in the DTG curve of the inclusion complex obtained by CO; this indicates the higher probability of replacing water molecules in the β-CD cavity with the monoterpene. According to Mura et al. [44], this can be explained by favoring hydrophobic interactions between POH and the nonpolar cavity of the β-CD resulting in a lower energy state, which favors the formation of the new structure. Then, a second, more pronounced mass loss event was seen, which corresponds to the degradation of β-CD. The data obtained by TG are in agreement with those recorded by DSC (Figure S1, Supplementary material).

**Figure 2:**
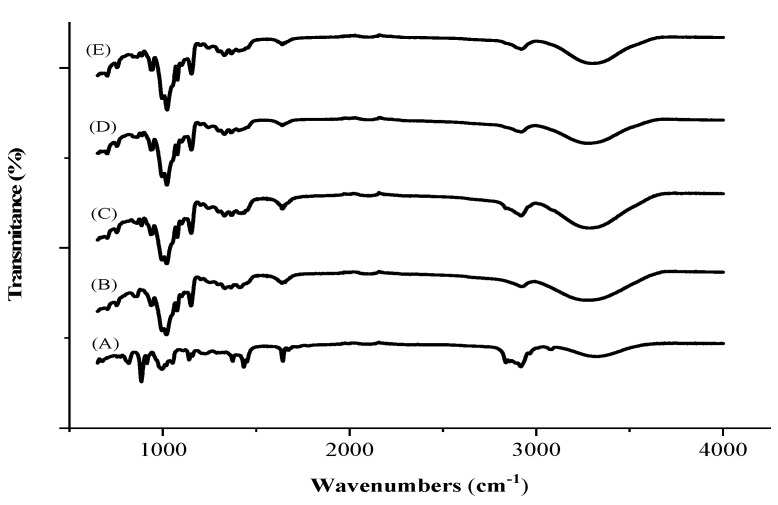
Fourier transform spectrophotometry (**A**) Perillyl alcohol, (**B**) β-cyclodextrin, (**C**) Co-evaporation, (**D**) Malaxation and (**E**) Physical mixture.

FTIR is useful to monitor the formation of an inclusion complex in the sample [45]. Figure 2 displays the FTIR spectra of POH, β-CD and complexes obtained by PM, MA and CO. POH exhibited typical OH (free) axial deformations bands, characteristic of the hydroxyl group at 3596.8 cm^−1^, alkene CH at 3009.8 cm^−1^ and C=C at 1667.9 cm^−1^. Additionally, the bands observed in the 1500–1300 cm^−1^ region correspond to the C–C stretch absorption bands of the ring. The absorption bands β-CD shown between 3718–3072 cm^−1^ are reference OH group vibration and a strong band is seen from 3000 cm^−1^ to 3300 cm^−1^, resulting from the OH group stretching, which suggests water loss and subsequent hydrogen bond breakage and POH inclusion. Similar findings were reported by Trindade et al. [21], who also verified cyclic structure C–H at 2800 cm^−1^, C–O stretch bands at 900 cm^−1^ and C=C at 1604 cm^−1^ and 1375 cm^−1^. PM spectra showed an overlap of the individual POH and β-CD bands, suggesting a weak interaction between POH and β-CD. Analyzing inclusion complexes spectra from MA and CO samples, there is an O–H axial deformation characterized by a narrow absorption band around 3500–3000 cm^−1^. Changes in intensity at this broad peak was associated with inclusion complex formation towards lower wavenumbers and decrease in intensity and broadening, upon complexation [46]. Also, a significant reduction in the POH characteristic absorption bands occurs probably due to a vibration restriction following the complexation process. The inclusion complex showed small differences in comparison to that of PM and the raw materials, indicating the presence of a weakly bound, as a hydrogen bond [47].

**Figure 3:**
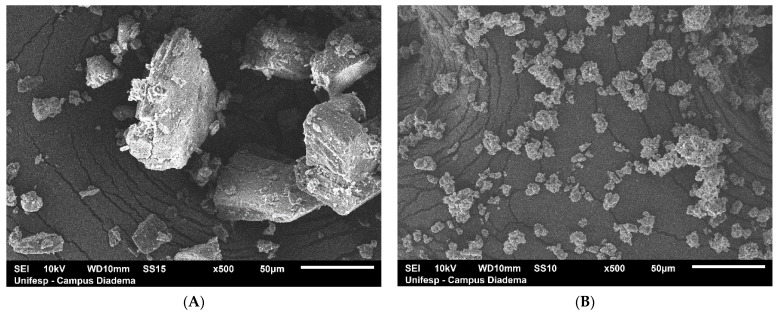
Scanning electron microscopy (SEM) images of (**A**) β-cyclodextrin (β-CD), (**B**) Co-evaporation.

Figure 3 shows the SEM of the β-CD and the β-CD:POH (1:1) produced by CO methodology at 500× magnification. Pure β-CD appears as crystalline particles of different sizes without a defined shape. The inclusion complex showed a significant reduction of particles, resulting in compact and homogeneous agglomerates which indicate a modification of the crystal and the powder. When the POH is complexed in the β-CD cavity, the crystallinity of the complex resulted in a more amorphous character. SEM results show the homogenous β-CD crystal after the POH inclusion. Similar results were also obtained with other monoterpenes, as α-terpineol [35], carvacrol [48] and citronellal [49].

**Figure 4:**
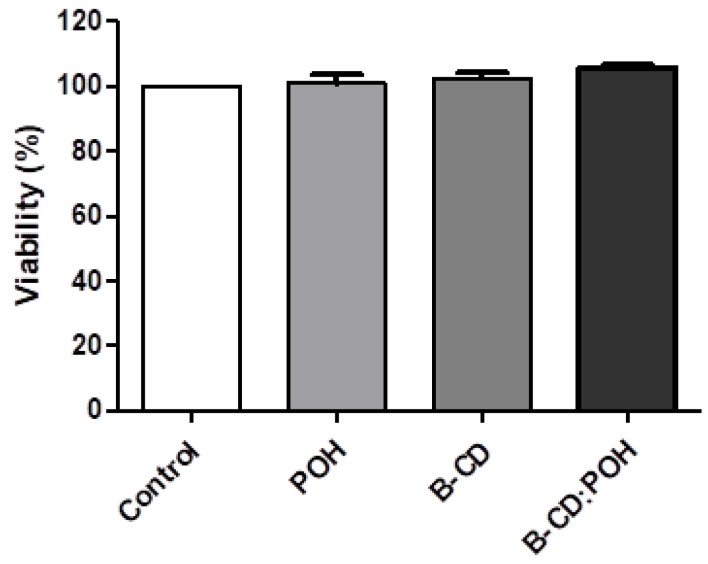
Effect of perillyl alcohol (POH), β-cyclodextrin and β-CD: POH (1:1) on the viability of human L929 fibroblasts determined by the MTT assay after 24 h of incubation. The negative control (C) was treated with the vehicle used to dissolve the drug (DMSO 5%). The data correspond to the mean ± S.E.M. of four independent experiments.

The results obtained in the present work indicate that for POH and for the inclusion complexes containing or not the monoterpene, resulted in 100% viability of human L929 fibroblasts after 24 h of incubation (Figure 4). These data suggest that both the isolated compound and the complex form are non-toxic to non-malignant cells, further demanding its evaluation in in vivo models.

Antitumor therapy encounters serious adverse effects due to the nonspecific action of antineoplastic drugs, which can trigger damage both in tumor cells and in healthy cells, compromising the homeostasis of healthy tissues [50]. Antineoplastic drugs induce a variety of metabolic damages to the patient, such as gastrointestinal disorders, nausea and immunosuppression, rashes, arthralgia, adynamia, neuro and nephrotoxicity, among countless others, in addition to the development of clinical resistance [51]. Thus, the search for more selective, effective and/or less aggressive drugs to reduce these side effects is essential for a successful antineoplastic therapy. An increasingly growing interest in the search for products for anticancer therapy has been seen to promote the elimination or inhibition of tumor growth [52]. The potential antineoplastic activity of the complex has been evaluated in a sarcoma 180 (S180) bearing mice. This model has the advantage of being easily handled for the implantation protocol, tumor growth up to 90% to 100% and rapid tumor evolution, which reduces the experimentation time [39].

**Figure 5:**
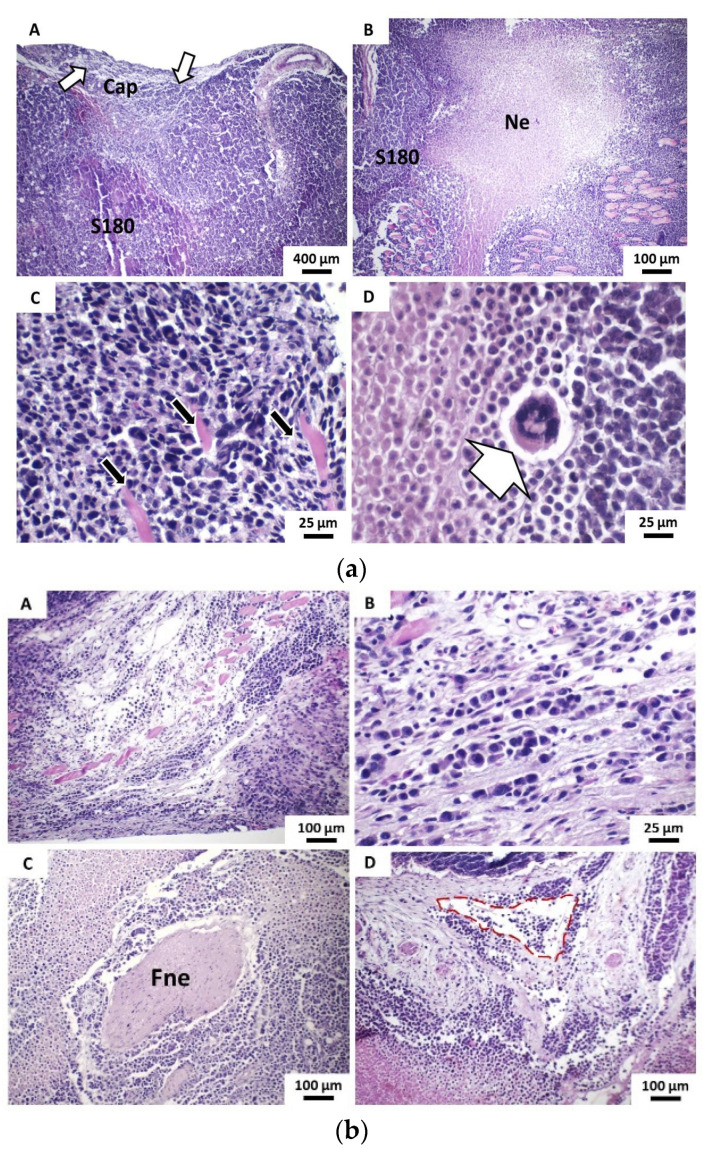

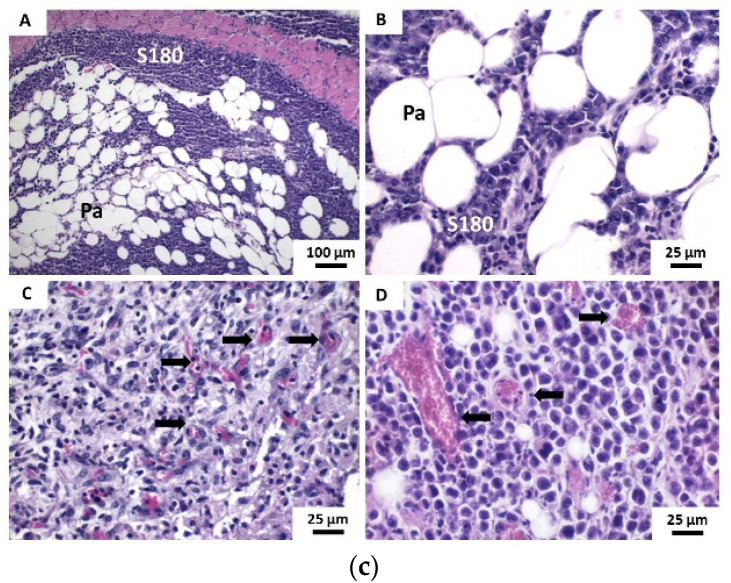
(**a**) Photomicrographs of histochemical technique (HE)-stained histological sections show the main histopathological features of tumors developed in the Vehicle group from the implantation of S100 cells. (A) The dermal superficial margin of the tumor shows discontinuous pseudocapsule (Cap) showing evident neoplastic infiltration (white arrows) (40×) and (B) extensive areas of coagulative necrosis (Ne) within the neoplastic cell blocks (40×). (C) Detail of tumor cells exhibit intense atypia and promote dissociation and destruction of striated skeletal muscle fibers (black arrows) (400×) and (D) Bizarre giant tumor cell (400×); (**b**) (A) Neoplastic cells promote infiltration and massive destruction of striated skeletal muscle fiber bundles (100×), (B) Strongly dissociated tumor cells arranged in “Indian row” cords (400×). (C) Neoplastic cells promote perineural invasion of peripheral nerve fibers (Fne) (100×). (D) Neoplastic cells observed inside the lymphatic vessel form a tumoral embolus (red outline) (100×) and (**c**) (A) and (B) Neoplastic cells (S180) promote massive infiltration of the hypodermic adipose panicle (Pa) (100× and 400×, respectively). (C) Intense capillary vascular neoformation (black arrows) on the periphery of the tumor and (D) in intraparenchymal areas (400×).

The histological analysis of tumors observed in the vehicle group were characterized by the formation of sheets and solid blocks of neoplastic cells, which deeply infiltrated the dermo-hypodermic tissue. In the dermal portion of the tumor, a discontinuous pseudocapsule was formed by loose fibrous connective tissue, with inflammatory reaction and moderate-intensity interstitial edema and infiltrated by neoplastic cells. The tumors exhibited extensive areas of intraparenchymal coagulative necrosis, forming large masses of irregularly shaped borders, which, in general, occupied more than 50% of the tumor area. Individually, parenchymal cells showed strong atypia, expressed by nuclear hyperchromatism and cellular pleomorphism, while unusual giant tumor cells were relatively frequent findings (Figure 5a). Also, mitotic activity was abundant, with the presence of typical and atypical mitosis figures. The infiltration of deep muscle planes is also quite evident; often, the tumor cells exhibited strong dissociation and were arranged in thin strands like the “Indian row”. Areas of perineural and lymphovascular invasion (formation of neoplastic cell emboli) were eventually observed in only two cases (Figure 5b). The massive infiltration of the hypodermic adipose panicle by neoplastic cells was also shown to be a frequent morphological finding. It was possible to evidence areas of neoformation of irregular capillary vessels, many of them hyperemic, both on the periphery of the tumor and inside the solid sheets of neoplastic cells. The inflammatory infiltrates present, albeit in mild to moderate intensity, were composed of lymphocytes and histiocytes, usually concentrated on the tumor margins and polymorphonuclear neutrophils, the latter particularly evident in the perinecrotic areas (Figure 5c).

**Figure 6:**
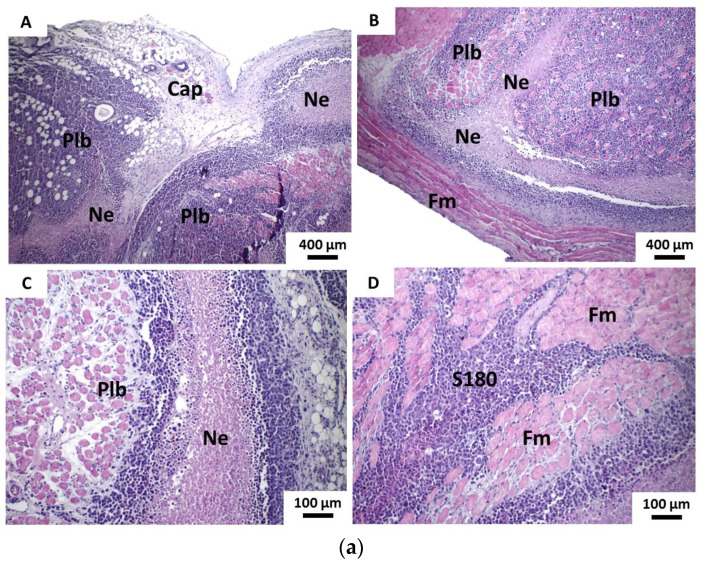

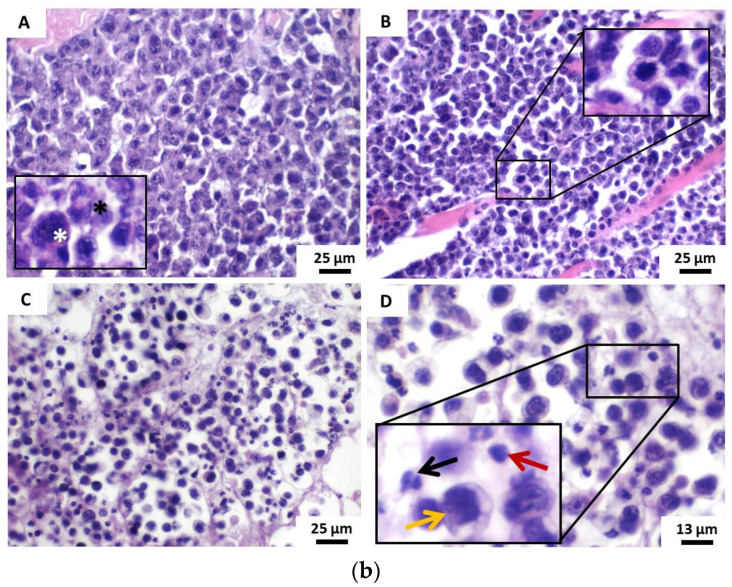
Photomicrographs of histological sections stained in HE show the main histopathological features of tumors developed in the 5-FU group from the implantation of S180 cells. (**a**) (A) The superficial area shows pseudocapsule and blocks of tumor cells separated by necrotic tissue trabecular, giving a pseudolobular aspect (40×). (B) Deep portion facing the hypodermis shows, in a similar way, the necrotic trabecular that separate blocks of tumor cells into pseudolobules; note the marked delimitation of the tumor by striated skeletal muscle fibers (40×). (C) Detail of the narrow bands of necrotic tissue (trabic) separating the pseudolobules (100 ×). (D) The central portion of the tumor shows tumor cells that invade bundles of muscle fibers, separating them into relatively well-preserved blocks (100×). Caption: Cap—superficial pseudocapsule; Coagulative necrosis; Plb—pseudolobules; Fm—skeletal striated muscle fibers; S180—viable sarcoma 180 tumor cells and (**b**) (A) Tumor cells showing mild to moderate atypia, with cells sometimes showing nuclei of chromatin, sometimes dense (white asterisk), sometimes dispersed (black asterisk) (400 ×, detail in 800 ×). (B) Figure of typical mitosis in prophase (400×, detail in 800×). (C,D) Intense inflammatory infiltrate and interstitial edema in the middle of the tumor parenchyma (400 × and 800 ×, respectively; details in 1500 ×). Legend: Yellow arrow—Tumor cell; Black arrow—neutrophil polymorphonuclear; Red arrow—lymphocyte.

5-FU group formed solid blocks of neoplastic cells exhibiting moderate atypia, still showing a balance between the number of cells with dense nuclear chromatin (hyperchromatic) and those with finely dispersed chromatin and sometimes with one to two prominent nucleoli. Tumor cells insinuated themselves in the connective tissue and invaded the skeletal striated musculature, sometimes severely dissociating the muscle bundles and adipose panicle and, at other times, separating them into compact and relatively well-preserved blocks or lobes, which denoted less invasive capacity/infiltration of tumor parenchymal cells. The areas of coagulative necrosis were less evident and are presented in the form of thin depicting a pseudolobular architectural aspect. There was also the presence of a peripheral fibrous pseudocapsule, well-marked but thin and discontinuous (Figure 6a). The mitotic activity proved to be infrequent and composed of a typical structure. Areas of intense intratumoral inflammatory infiltrate, rich in lymphocytes, macrophages and neutrophils were also evident. Moreover, the inflammatory infiltrate was also intense in the marginal tumor areas, especially in the pseudocapsule (peripheral fibrous connective tissue, particularly visible in the superficial portion of the tumors) (Figure 6b).

**Figure 7:**
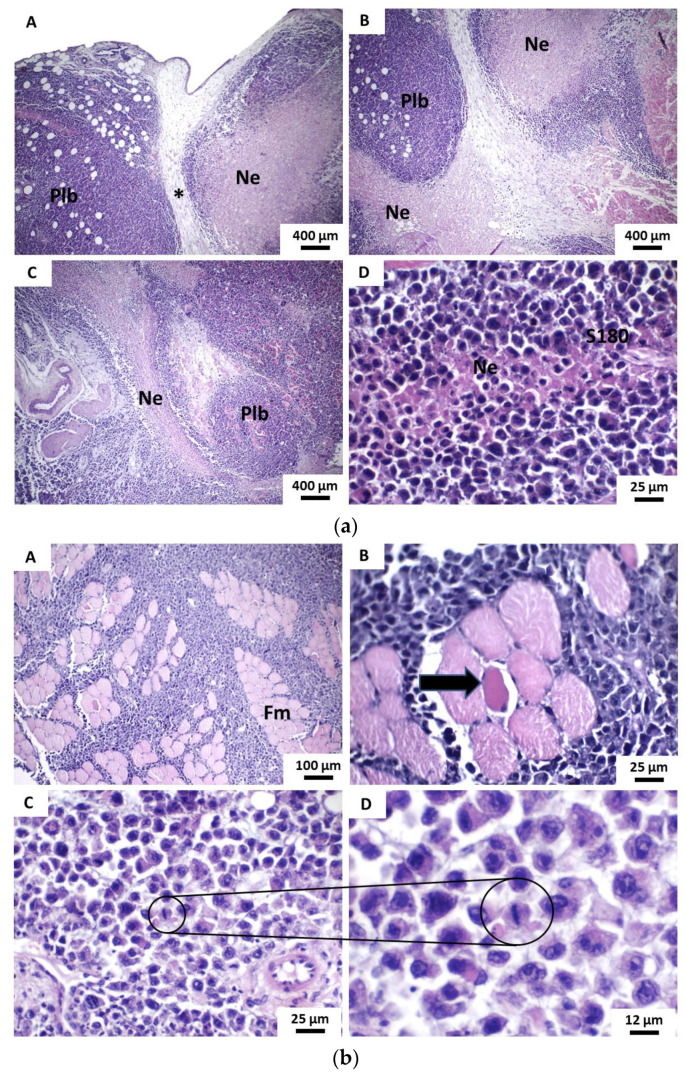
Photomicrographs of histological sections stained in HE show the main histopathological features of tumors developed in the β-CD:POH 50 mg/Kg group from the implantation of S180 cells. (**a**) (A–C) Tumor cells organized in a pseudolobular pattern (sometimes septated by fibrous connective tissue and sometimes by irregular bands of necrotic tissue) (40×). (D) Small necrotic tissue trabic amid tumor sheet cells (400 ×). Caption: Coagulative necrosis; Plb—pseudolobules; S180—viable sarcoma 180 tumor cells and (**b**)(A) Tumor cells infiltrate and dissociate solid blocks of muscle fibers (Fm) (100×). (B) Apoptosis of muscle cell (black arrow) in response to tumor infiltration (400×). (C) Atypical tumor cells showing low cohesivity and mitotic activity (400×). (D) Detail mitotic figure of tumor cell in metaphasis (800×).

POH/β-CD 50 mg/Kg group presented histological characteristics similar to those evidenced in the 5-FU group, expressed by an architectural arrangement of tumor cells also in irregular pseudolobules, now separated by edematous fibrous connective tissue and rich in inflammatory cells, sometimes separated by necrotic trabeculae tissue. It was observed that the proportion of areas of coagulative necrosis shown in this group was higher than in the 5-FU group (Figure 7a). The invasion pattern of striated skeletal muscle tissue and subcutaneous adipose panicle by tumor cells was also similar to the 5-FU group, with tissue dissociation in dense and compact blocks of muscle fibers and fat cells. Mitotic activity was still marked but seldom atypical mitosis (Figure 7b).

**Figure 8:**
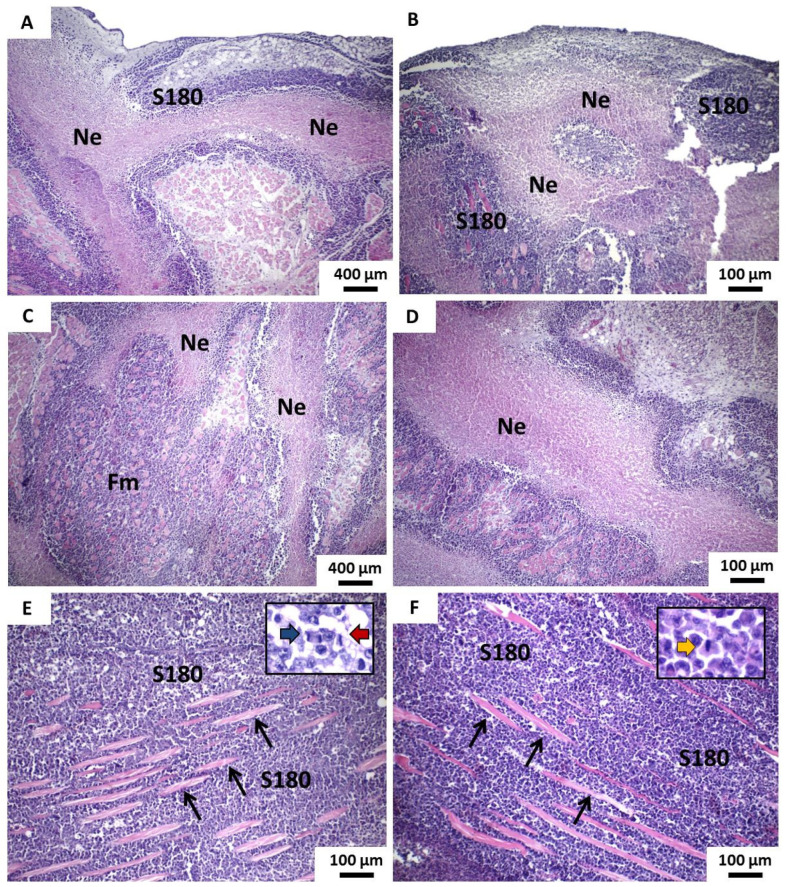
Photomicrographs of HE stained histological sections show the main histopathological features of tumors developed in groups β-CD:POH (100 and 200 mg/Kg) from the implantation of S180 cells. (**A**,**B**) Irregular tumor boundaries and areas of peripheral coagulative necrosis and (**C**,**D**), as well as extensive blocks of necrosis in the most central areas of the tumor masses observed in groups β-CD:POH 100 mg/Kg and 200 mg/Kg, respectively (100×). (**E**) Intense dissociation of muscle fibers by tumor cells in both groups β-CD:POH 100 mg/Kg and (**F**) β-CD:POH 200 mg/Kg (100×). In the details, there are figures of mitosis in different phases of the cell cycle (400×). Legend: S180—Viable tumor cells of sarcoma 180; Coagulative necrosis; Thin black arrows—striated skeletal muscle fibers; Blue arrow—anaphase mitosis; Red arrow—metaphase mitosis; Orange arrow—prophase mitosis.

POH/β-CD 100 and 200 mg/Kg groups presented similar histological characteristics, characterized by more dense neoplastic proliferation, without lobulation or apparent pseudolobulation, with areas of extensive necrosis both within the tumor mass and in the marginal areas. The inflammatory reaction was limited to tumor margins and perinecrotic areas. The tumoral boundaries were shown to be irregular and strong dissociation and destruction of striated skeletal muscle fibers were observed. Besides, mitotic activity was expressive but with rare atypical (Figure 8). The degree of cytological atypia proved to be highly variable between the various tumors, sometimes milder and sometimes more intense; however, giant cells were rarely seen while morphological signs of the perineural or vascular invasion were not observed.

Concerning the histopathological results, the treatment with POH/β-CD at a dose of 50 mg/kg promoted approximately 60% inhibition of tumor growth in mice. The mechanisms of action that determine this biological activity are not yet fully elucidated but there are indications, in the literature, of direct and indirect action by the POH on malignant neoplasms. It has been shown that POH promotes inhibition of the Ras signaling pathway and, consequently, cell proliferation and apoptosis induction in the phase of promoting dermal carcinogenesis [53]. On the other hand, it has recently been reported that POH can block the transition of the phenotype of undifferentiated mesenchymal cells into endothelial cells (mesenchymal-endothelial transition or TMEnd) derived from human multiform glioblastomas [3]. The authors believe that, as EndTM is essential for the development of tumor angiogenesis phenomena, supporting neoplastic nutrition, this inhibitory effect could, indirectly, inhibit tumor progression and recurrence. However, further studies are needed to prove.

It should also be noted that the intensity of tumor inhibition of the POH/β-CD (50 mg/kg) was similar to that described by Andrade et al. [37], using free POH in a dose 4 times greater (200 mg/kg). These data suggest that the technological strategy of complexing this monoterpene into β-cyclodextrin inclusion complexes (POH/β-CD) not only solves the problems related to the volatility of the compound but also increases its efficiency as an antitumor agent, as it allows obtaining similar results at much lower doses. This increase in the pharmacological activity of bioactive compounds after inclusion into cyclodextrin complexes is well defined in the literature [30] and supports the results obtained in our study.

Besides, the groups treated with 5-FU and POH/β-CD (50 mg/kg) presented distinct morphological characteristics compared to others. The lower degree of tumor necrosis, less mitotic activity and better definition of tumor borders, sometimes showing areas of peritumoral fibrosis. The mitotic index provides evidence about tumor proliferative activity; the formation of areas of necrosis is often associated with cell proliferation so intense and rapid that neovascularization does not occur with the speed necessary to provide nutrition to tumor cells and, thus, suggesting high proliferative activity; lymphocytic peritumoral fibrosis provide evidence about the interaction between tumor and host tissues [54]. Thus, it is possible to speculate that such findings, almost not observed in the other groups, could be related to a less aggressive biological behavior of tumors treated with 5-FU and POH/β-CD (50 mg/kg).

Tumor proliferation was analyzed using in situ immunostaining of the Ki67 antigen. This protein is expressed in the cell nucleus in all phases of the cell cycle (G1, S, G2 and M), is widely used as an indicator of cell proliferation and even tumor prognosis [55,56]. In this sense, tumors with high proliferative indices (strongly positive for the Ki67 antigen) would show more aggressive biological behavior, the opposite being true for those with lower proliferative indices (showing weak positivity for Ki67).

**Figure 9:**
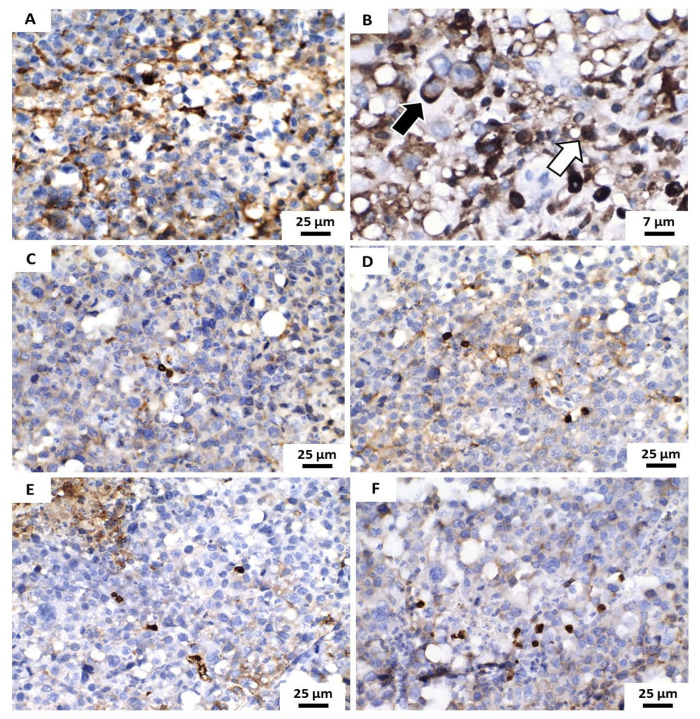
Photomicrographs of histological sections demonstrate the immunohistochemical expression of the Ki67 antigen (brownish color) in the analyzed tumors. (**A**,**B**) Vehicle Group shows tumor cells showed predominantly nuclear positivity (light arrow) and eventually, cytoplasmic immunoexpression of this antigen (dark arrow). (**C**–**F**) represent the groups treated with 5-FU (25 mg/Kg/day) and 50, 100 and 200 mg/Kg/day of the formulation contain POH/β-CD inclusion complex.

Immunostaining for Ki67 antigen was observed in all analyzed samples but the immunostained cells varied significantly between specimens. Although immunohistochemical positivity has been evidenced mainly in the cell nucleus, in many cases the cytoplasmic/membranous expression of this antigen has also been identified in tumor cells. However, only nuclear immunostaining was considered for quantitative analysis of the proliferative tumor index. The distinct morphological characteristics suggest a less aggressive tumor behavior in the groups treated with 5-FU and POH/β-CD (50 mg/kg) (Figure 9).

In the present study, immunostaining for the Ki67 antigen was evidenced predominantly in the nuclei, corroborating the classic pattern of immunoexpression described in previous studies with sarcoma 180 [38]. However, eventually and surprisingly, focal areas of cytoplasmic/membranous immunostaining of this antigen were also found. This atypical marking pattern for the Ki67 antigen has been described previously [57] but the mechanisms behind this phenomenon and its real clinical significance remain unclear. It has been suggested that this pattern of immunostaining would occur for three possible reasons: (i) an alleged cross-reaction with other cytoplasmic antigens similar to the histone ki67 nuclear protein; (ii) technical artefacts and (iii) possible relocation of the Ki67 protein from the nucleus to the cytoplasm during the cell cycle [58].

In our work, nuclear immunoexpression of the Ki67 antigen was reduced in the groups treated with 5-FU and POH/β-CD (50 mg/Kg) compared to the group treated with the vehicle. These data suggest a significant reduction of cycling cells (in proliferation) and seem to agree with the results obtained in both macroscopic analyses of the inhibition of neoplastic growth and in the study of the histological characteristics of experimental tumors. On the other hand, the mechanisms that determine these results have yet to be investigated, as they could result both from the possible blockage of the cell cycle and from the increase in death due to apoptosis of cycling cells. It is important to note that, since the areas of coagulative necrosis were much less extensive in these two groups (5FU and POH/β-CD (50 mg/Kg), it is possible that speculation that the smallest tumor growth by inducing a higher rate of necrosis is unlikely. Thus, further studies are needed in order to elucidate this issue.

## 4. Conclusions

In this work, perillyl alcohol/β-cyclodextrin (1:1) inclusion complexes have been successfully obtained. Three distinct complexation approaches have been adopted, while molecular docking confirmed the hydrogen-bond interaction between β-cyclodextrin. The best interaction was seen for the complexes obtained by co-evaporation. The formation of this inclusion complex was also demonstrated by SEM, as a homogenous β-CD crystal was obtained after the POH inclusion, in contrast to the pure β-CD. The inclusion of POH in β-cyclodextrin resulted in 100% of cell viability after 24 h against human fibroblast line L929. From the histopathological analysis, the in vivo studies in S180-induced mice treated with POH/β-CD at a dose of 50 mg/kg demonstrated the inhibition of tumor growth about 60%. In situ immunostaining of the Ki67 antigen was used to analyze tumor proliferation also showing a significant reduction of cycling cells. Our results anticipate the potential advantages of using monoterpene of essential oil—perillyl alcohol—as an anti-tumor drug, emphasizing the added value of CD-docking as an approach to improve the drug’s performance in vitro and in vivo, while reducing the risk of volatility.

## Figures and Tables

**Table 1:** Molecular docking affinity energy and interaction groups calculated using AutoDock vinna for the POH/βCD complex.

Compound	Affinity (kcal/mol)	Interaction	Interaction Type	From	To	Distance (Å)
Perillyl Alcohol	−4.0	O	Hydrogen Bond	Perillyl Alcohol (OH in C10)	βCD (OH in C6)	2.23

## Data Availability

The data presented in this study are available on request from the corresponding authors.

## Supplementary Materials

The following are available online at https://www.mdpi.com/1999-4923/13/2/245/s1, Figure S1: DSC thermogram (A) Perillyl alcohol, (B) β-cyclodextrin, (C) Co-evaporation, (D) Malaxation and (E) Physi-cal mixture, Figure S2: TG/DTGG curves of (A) Perillyl alcohol, (B) β-cyclodextrin, (C) Co-evaporation, (D) Malaxation and (E) Physical mixture.
