# Supplementary material for: Anti-Tumor Efficiency of Perillylalcohol/β-Cyclodextrin Inclusion Complexes in a Sarcoma S180-Induced Mice Model
Source: Pharmaceutics. 2021 Feb 10;13(2):245. doi: 10.3390/pharmaceutics13020245 (PMC7916601; doi:10.3390/pharmaceutics13020245)
Supplement: Supplementary file 1 [file pharmaceutics-13-00245-s001.pdf]

pharmaceutics

Supplementary Material

Anti-Tumour Efficiency of Perillylalcohol/β-Cyclodextrin Inclusion Complexes in a Sarcoma S180-Induced Mice Model

Allan A. Rezende, Rafael S. Santos, Luciana N. Andrade, Ricardo G. Amaral, Matheus Pereira, *Cristiane Bani*, Mo Chen, Ronny Priefer, Classius F. da Silva, Ricardo L. C. de Albuquerque Junior, Eliana B. Souto[8], Patrícia Severino

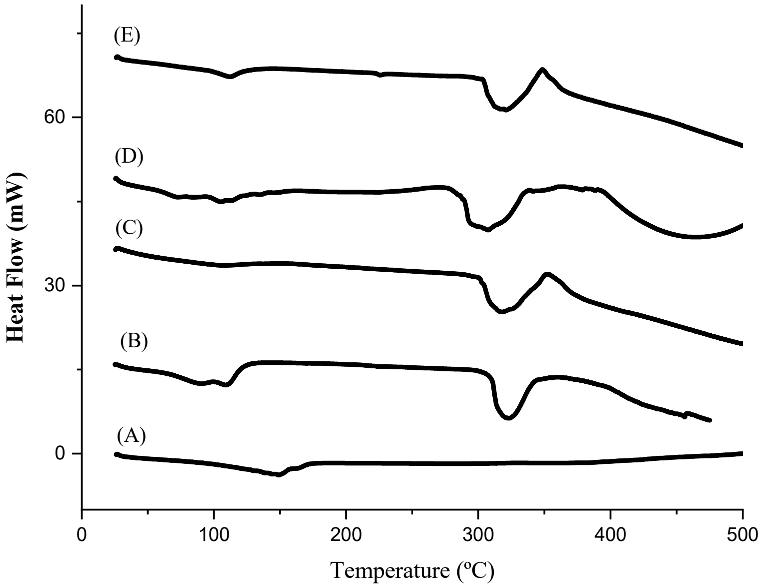

Figure S1. DSC thermogram (**A**) Perillyl alcohol, (**B**) β-cyclodextrin, (**C**) Co-evaporation, (**D**) Malaxation and (**E**) Physical mixture.

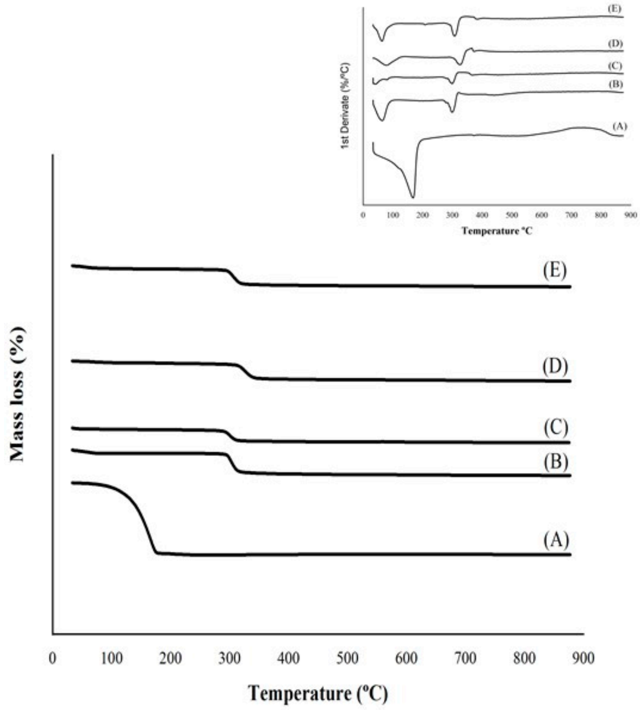

Figure S2. TG/DTGG curves of (**A)** Perillyl alcohol, (**B**) β-cyclodextrin, (**C**) Co-evaporation, (**D**) Malaxation and (**E**) Physical mixture.
